# Patient-derived organoids as a platform for drug screening in metastatic colorectal cancer

**DOI:** 10.3389/fbioe.2023.1190637

**Published:** 2023-05-22

**Authors:** Xingfeng He, Yan Jiang, Long Zhang, Yaqi Li, Xiang Hu, Guoqiang Hua, Sanjun Cai, Shaobo Mo, Junjie Peng

**Affiliations:** ^1^ Department of Colorectal Surgery, Fudan University Shanghai Cancer Center, Fudan University, Shanghai, China; ^2^ Department of Oncology, Shanghai Medical College, Fudan University, Shanghai, China; ^3^ Department of Nursing Administration, Fudan University Shanghai Cancer Center, Fudan University, Shanghai, China; ^4^ Department of Radiation Oncology, Fudan University Shanghai Cancer Center, Shanghai, China

**Keywords:** metastatic colorectal cancer, patient-derived organoids, preclinical model, drug screening, personalized therapy

## Abstract

**Introduction:** Most advanced colorectal cancers are aggressive, and there is a lack of effective methods for selecting appropriate anticancer regimens. Patient-derived organoids (PDOs) have emerged as preclinical platforms for modeling clinical responses to cancer therapy.

**Methods:** In this study, we successfully constructed a living biobank with 42 organoids derived from primary and metastatic lesions of metastatic colorectal cancer patients. Tumor tissue was obtained from patients undergoing surgical resection of the primary or metastatic lesion and then used to establish PDOs. Immunohistochemistry (IHC) and drug sensitivity assays were performed to analyze the properties of these organoids.

**Results:** The mCRC organoids were successfully established with an 80% success rate. The PDOs maintained the genetic and phenotypic heterogeneity of their parental tumors. The IC50 values of5-fluorouracil (5-FU), oxaliplatin, and irinotecan (CPT11) were determined for mCRC organoids using drug sensitivity assays. The in vitro chemosensitivity data revealed the potential value of PDOs for clinical applications in predicting chemotherapy response and clinical outcomes in mCRC patients.

**Discussion:** In summary, the PDO model is an effective platform for in vitro assessment of patient-specific drug sensitivity, which can guide personalized treatment decisions for patients with end-stage CRC.

## Introduction

Colorectal cancer (CRC) is the third most common cancer worldwide, and its incidence and mortality continue to increase (Sung et al., 2021). Approximately 20% of patients with newly diagnosed CRC have synchronous metastases, of which the liver is the most commonly affected organ (van der Geest et al., 2015), and nearly 50% of initially localized patients will develop metastases after curative treatment (Ciardiello et al., 2022). As a result of systematic treatment strategies, including surgery, chemotherapy, radiation, and immunotherapy, the median overall survival of stage IV CRC has significantly improved over the past decades (Van Cutsem et al., 2016). However, despite improvements in cancer therapy, responses to currently available therapeutics vary considerably across patients due to tumor heterogeneity and different levels of drug resistance (Guinney et al., 2015; Punt, Koopman, and Vermeulen, 2017; Roerink et al., 2018). Therefore, there is an urgent need for new methods to predict the efficacy of individualized treatment.

Historically, cancer cell lines and patient-derived tumor xenografts (PDTXs) have been used as preclinical models to assess the clinical response of potential drug candidates for personalized treatment. However, these models are limited in clinical application due to a number of shortcomings. Cancer cell lines are two-dimensional models, that usually do not have organ structures and cannot reflect tumor heterogeneity (Domcke et al., 2013). The establishment of PDTXs is often inefficient, time-consuming, and technically challenging (Tuveson and Clevers, 2019). In brief, there is a lack of novel preclinical models to predict the response to personalized cancer treatment. Therefore, PDOs are used to fill the gap in traditional preclinical models. PDOs can be constructed from tumor tissues with a high success rate, short culture period, and unlimited expansion, which can faithfully recapitulate the original tumor’s *in vivo* functionality, architecture, and genetic characterization (Lau et al., 2020). To date, PDO *in vitro* models have been developed as a high throughput system for drug testing (Boj et al., 2015; van de Wetering et al., 2015; Weeber et al., 2015; Boehnke et al., 2016).

In the present study, we established a living biobank of organoids derived from CRC patients, and we explored the application prospect of PDOs in mCRC as a preclinical model of precision cancer medicine. We also evaluated whether mCRC PDOs could effectively predict patient drug response in clinical practice.

## Materials and methods

### Human specimens

The Institutional Review Boards of Fudan University Shanghai Cancer Center approved this study of human tumor samples. Tumor tissues of mCRC patients were obtained from patients who underwent surgical resection in the Department of Colorectal Surgery, Fudan University Shanghai Cancer Center. Postoperative clinical data of each CRC patient were collected from the medical record system, including sex, age, tumor size, clinical stage, and computed tomography (CT) or magnetic resonance imaging (MRI) data. This study followed the accepted ethical guidelines (the Declaration of Helsinki). Informed consent was obtained from all of the participants.

### Tissue processing

The isolated tumor tissues were preserved in DMEM medium (GIBCO) and transferred to the laboratory. The tissues were washed in cold PBS with penicillin/streptomycin (Solarbio) three times for 5 min each. The washed tissue was moved to a 10-cm cell culture dish, part of the tissue was sectioned for wax block making, and the remaining tissue was finely minced and then transferred to a centrifuge tube. After washing with cold PBS 5 times for 5 min and centrifuging at 500 × *g* for 5 min, the tumor tissue precipitate was collected and digested in 8 mL preheated digestive solution at 37°C for 30–60 min. The formula of the digestion solution was 7 mL DMEM medium, 20 mg/mL hyaluronidase (Solarbio), 1.5 mg/mL collagenase II (Solarbio), 0.1 mg/mL dispasetype II (Sigma-Aldrich), 10 mM RHOK inhibitor y27632 (Sigma-Aldrich), and 500 U/mL collagenase IV (Sigma-Aldrich). At the end of digestion, the tissues were filtered through a 100 μm cell strainer and then centrifuged at 500 × g for 5 min. The obtained precipitate was washed with cold PBS 5 times for 5 min. The organoid was resuspended in an appropriate amount of matrigel and embedded in a 24-well plate (Sorfa) at 50ul of matrigel (Corning) per well. Then we placed the 24-well plate in an incubator at 37°C for 10–15 min. After the matrigel was polymerized, 500 μL of organoid culture medium was added, and organoids were photographed at the proper times to record their growth status.

### Organoid culture

For tumor organoid passaging, the organoid culture medium was removed, and then the organoids were collected from the wells of a 24-well plate into a 15 mL centrifuge tube using 2–3 mL of cold PBS containing 0.1% BSA. The suspension was mixed with a 1-mL pipettor approximately 100 times, and the organoids were mechanically sheared through the pipette tip. Organoid precipitates were then collected by centrifugation at 500 × g for 5 min, and organoids were seeded in a 24-well plate as described above. The culture medium was changed every 3 days. For the cryopreserved organoids, the organoids in good condition were collected. After the matrix glue was removed, the organoids were resuspended in a cryopreservative medium containing no serum (CELLBANKER™ ^2^, ZENOAQ) and transferred to a cryopreserved tube for storage in liquid nitrogen.

The Complete human mCRC organoid culture medium composition was as follows, which was described previously (Mo et al., 2022): 1× Advanced DMEM/F12 medium (GIBCO), 500 ng/mL R-spondin 1 (Sino Biological Inc.), 100 ng/mL Noggin (Sino Biological Inc.), 50 ng/mL EGF (Sino Biological Inc.), 1× HEPES (GIBCO), 1× Glutamax (GIBCO), 1× Normocin (InvivoGen), 1× Gentamicin/amphotericin B (GIBCO), 1 × N2 (Invitrogen), 1 × B27 (Invitrogen), 1 mM N-Acetyl-L-cysteine (Sigma-Aldrich), 10 mM Nicotinamide (Sigma-Aldrich), 500 nM A-83-01 (Tocris), 3 μM SB202190 (Sigma-Aldrich), 10 nM Gastrin (Sigma-Aldrich) and 10 nM Prostaglandin E2 (Sigma-Aldrich).

### H&E and IHC staining

Tumor tissues and organoids were fixed in 10% neutral buffered formalin for 24 h, and the fixed tumor tissues and organoids were embedded in paraffin. The paraffin blocks were serially sectioned at a thickness of 4 μm and used for H&E and IHC staining. IHC staining for MSH2, PMS2, MLH1, MSH6, Ki-67, CDX2, CK20, β-catenin, and CK-pan (the concentrations of primary antibodies are listed in Supplementary Table S1) was performed for all tumor tissues and tumor organoids. The sections were treated with 3% hydrogen peroxide for 10 min at room temperature to block endogenous peroxidase activity. Then sections were incubated with EDTA antigen repair solution in vapor copper for 20 min and blocked with 10% donkey serum for 1 h, followed by incubation with primary antibody overnight at 4°C and secondary antibody (GTVision III Detection System/Mo & RB, Gene Tech, GK500710) for 1 h at room temperature. Images of H&E and IHC-stained sections were captured with by a Zeiss microscope (ZEISS, Imager. M2).

### Drug screen

Well-conditioned CRC organoids were seeded in 96-well cell culture plates (Corning). Approximately 100 organoids were implanted in 5 μL matrigel, and 200 μL medium was added to each well. The drug concentrations for 5-FU, CPT11 or oxaliplatin monotherapy were 50 μM, 25 μM, 10 μM, 5 μM, 1 μM, 0.5 μM, 0.1 μM, 0.01 μM, and 0 μM. For the FOLFOX regimen (5-Fu: leucovorin: oxaliplatin = 25: 5: 1), the FOLFIRI regimen (5-Fu: leucovorin: CPT11 = 25: 5: 2), and the FOLFIRINOX regimen (5-Fu: leucovorin: CPT11: oxaliplatin = 25: 5: 2: 1), the final concentration of 5Fu was maintained at 50 μM, 25 μM, 10 μM, 5 μM, 1 μM, 0.5 μM, 0.1 μM, 0.01 μM, and 0 μM, as described previously (Mo et al., 2022). Each drug concentration gradient contained three replicate wells to avoid bias. Three days later, the medium containing the specific drug concentration was updated. After 6 days of drug treatment, the viability of the organoids was measured by the CellTiter-Glo 3D Reagent (Promega, G9683) according to the manufacturer’s instructions, and organoid viability luminescence was detected in a multifunctional microplate reader (Molecular Devices, SpectraMax M5). The IC50 was plotted using GraphPad Prism 8 (LA Jolla, CA, United States), and IC50 values were calculated.

### Statistical analysis

Assessment of changes in tumor burden before and after treatment is an important feature of the clinical evaluation of cancer therapy. The RECIST guidelines (version 1.1) (Eisenhauer et al., 2009) were used to evaluate tumor response. In the correlation analysis between drug test results and treatment response of mCRC patients, patients with SD and PR were considered to be sensitive to chemotherapy (good response), while patients with PD were considered to be resistant to chemotherapy (poor response). All statistical analyses were performed using GraphPad Prism 8 (La Jolla, CA, United States).

## Results

### Establishing a collection of mCRC organoids

**FIGURE 1:**
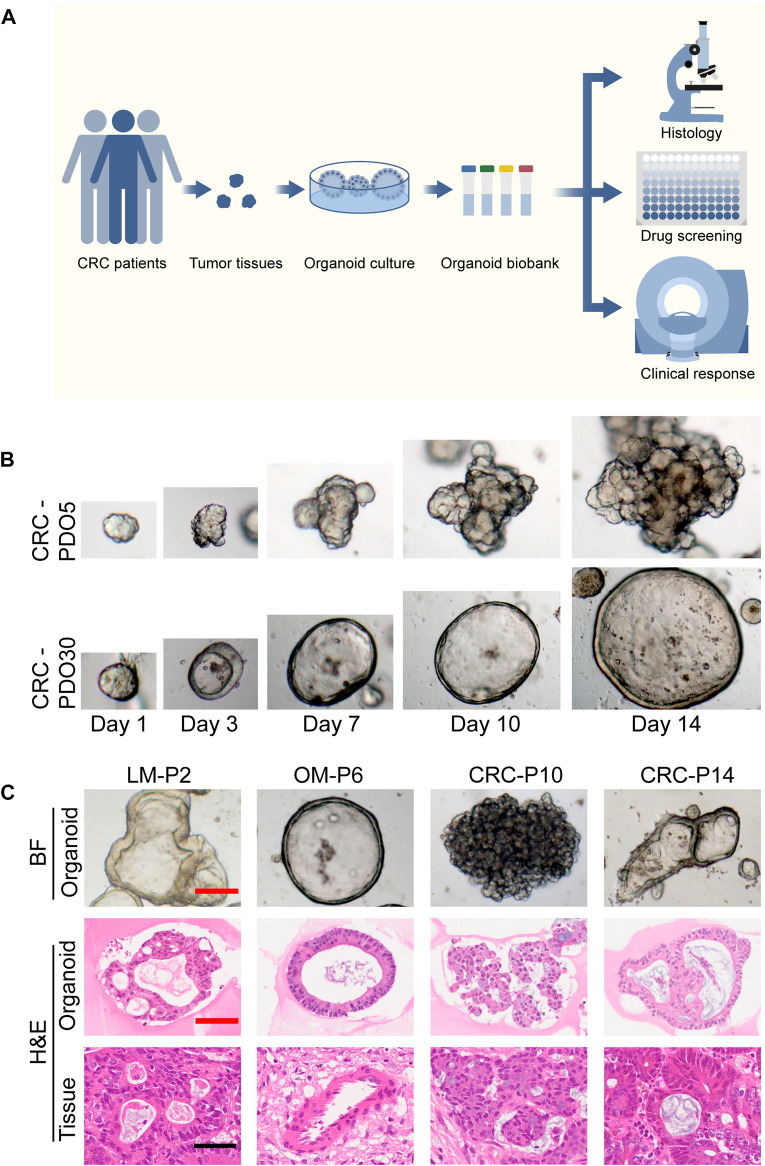
Establishment of a biobank of mCRC PDOs. **(A)** Flow diagram of the study, including the establishment of mCRC organoid lines, as well as the histological characterization, drug screen on organoids. **(B)** Time course culture of mCRC organoids with different morphologies (CRC-PDO5, irregular solid/compact structures; CRC-PDO30, thin-walled cystic structures). **(C)** Histopathological features of primary tumors and PDOs. H&E comparison of four CRC organoids (LM-P2, OM-P6, CRC-P10, and CRC-P14) with the corresponding tumor from which they were derived (4 mCRC patients). Representative images of these CRC organoids in bright-field were displayed (top). Black scale bar, 100 μm. Red scale bar, 50 μm. PDOs, patient-derived organoids; mCRC, metastatic colorectal cancer; LM, liver metastasis; OM, ovarian metastasis.

Freshly resected tumor tissues were processed by combining mechanical mincing and enzymatic digestion to obtain organoid cells. The acquired organoids were placed in Matrigel drops, and then 500 μL organoid medium was added to each well. Finally, we successfully established an mCRC PDO biobank *in vitro* from patients who underwent surgical resection in our center (Figure 1A). 52 surgical tissue samples were obtained, and 42 organoids were successfully cultured (success rate, 80.8%), including 24 primary CRC organoids, 15 metastatic liver organoids, 2 peritoneal metastatic organoids, and 1 ovarian metastatic organoid, which was in line with previous reports (Vlachogiannis et al., 2018; Lau et al., 2020). 42 organoid lines were derived from 36 patients, of whom the demographic and clinicopathological characteristics are presented in Supplementary Table S2. The most common pathological type was adenocarcinoma, and most tumors were moderately differentiated. These organoid lines were continuously passaged and propagated and have been successfully cryopreserved and thawed for regeneration. Seven organoid lines could not be successfully established due to bacterial contamination, and 3 organoid lines stopped growing after passage.

### Organoids show histological features similar to those of the original tumors

**FIGURE 2:**
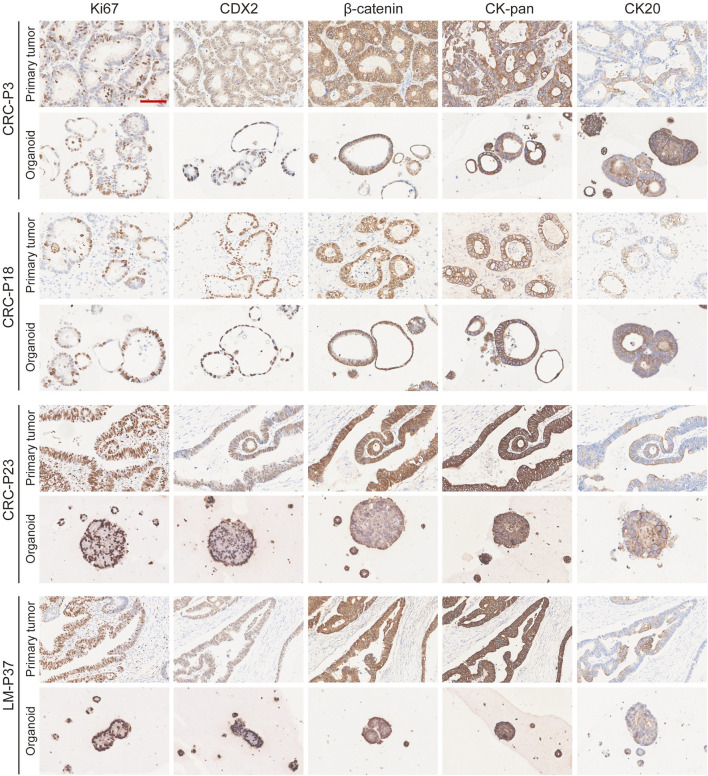
Marker expression analysis of mCRC organoids. mCRC PDOs derived from four patients (CRC-P3, CRC-P18, CRC-P23, and LM-P37) are compared to their primary tumors for ki-67, CDX2, β-catenin, CK-pan, and CK20 staining. Scale bar, 100 μm.

**FIGURE 3:**
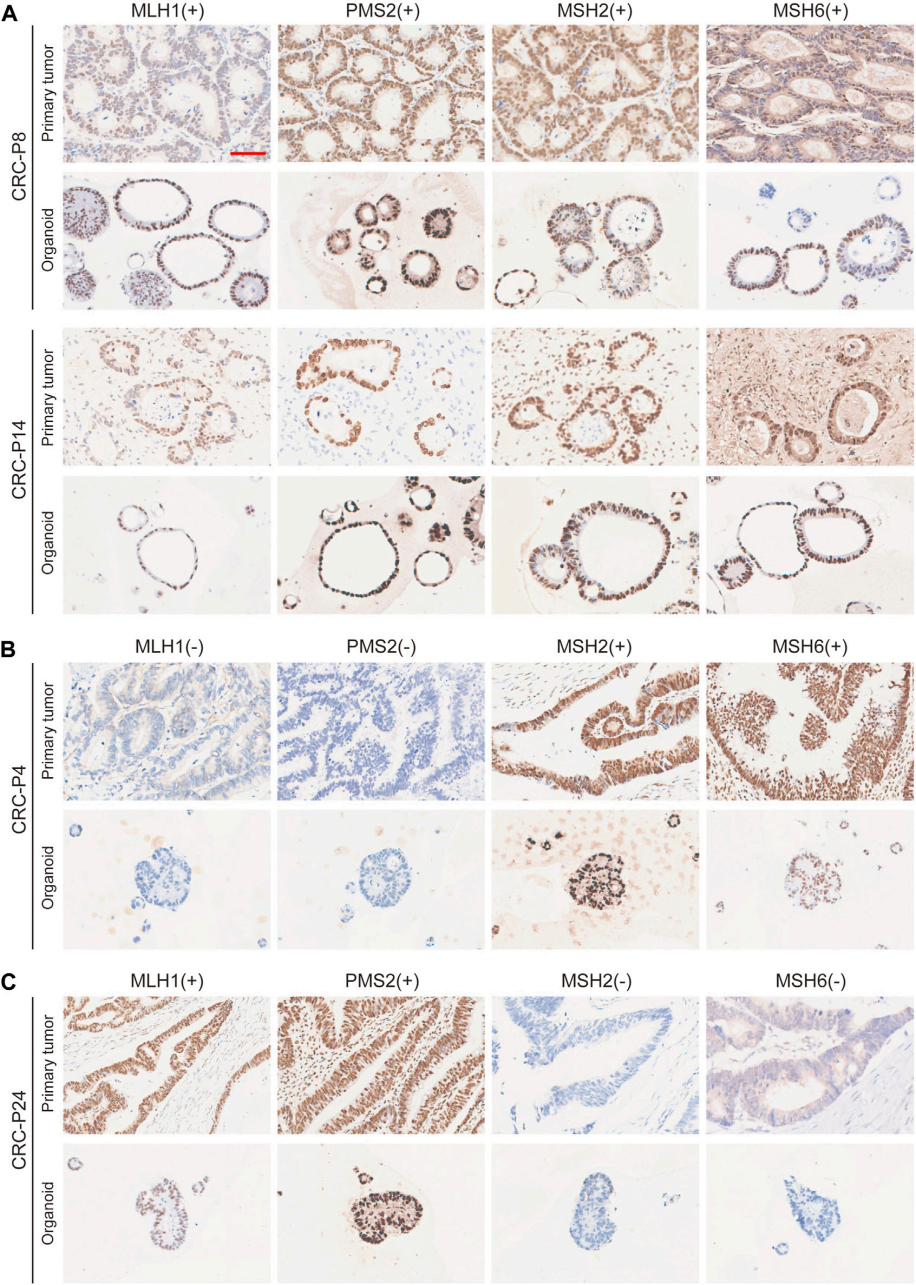
Comparison of Nuclear Mismatch Repair Proteins between mCRC Patient and PDO Samples. Immunohistochemistry of the nuclear mismatch repair (MMR) proteins MLH1, MSH6, MSH2, and PMS2. **(A)** Displayed are two patients with proficient MMR, CRC-P8 and CRC-P14, and two patients with deficient MMR, **(B)** CRC-P4, MLH1 (−) /PMS2 (−); **(C)** CRC-P24, MSH2 (−)/MSH6 (−). Scale bar, 100 μm.

The morphology of organoids with two typical characteristics is shown in Figure 1B, and we documented the time course of the formation of these two organoids after passage. CRC-PDO5 showed an irregular solid/compact structure, and CRC-PDO30 showed a thin-walled cystic structure. To investigate whether the histological characteristics of the parental tumors were preserved in the mCRC organoids, we performed histopathological analysis of H&E-stained tumor and organoid sections. Organoids retained histological features similar to the primary tumors from which they were derived (Figure 1C). Except for the histological conservation, subsequent expression analysis of signature molecules revealed similar staining patterns between organoids and the matched tumors for Ki67, CDX2, β-catenin, CK-pan, and CK20 (Figure 2)**.** These proteins have been considered potential markers for laboratory testing and clinical diagnosis of CRC. Similarly, we detected the expression levels of MMR-related proteins (MLH1, MSH6, MSH2, and PMS2). CRC-P8 and CRC-P14 showed a pMMR status (Figure 3A). CRC-P4 was dMMR with the loss of MLH1 and PMS2 (Figure 3B). CRC-P24 was pMMR with the loss of MSH2 and MSH6 (Figure 3C). In brief, PDOs and parental tumors have similar histopathological architecture, thus preserving the identity of the individual of origin.

### Organoids as a platform to test the drug sensitivity of tumors

**FIGURE 4:**
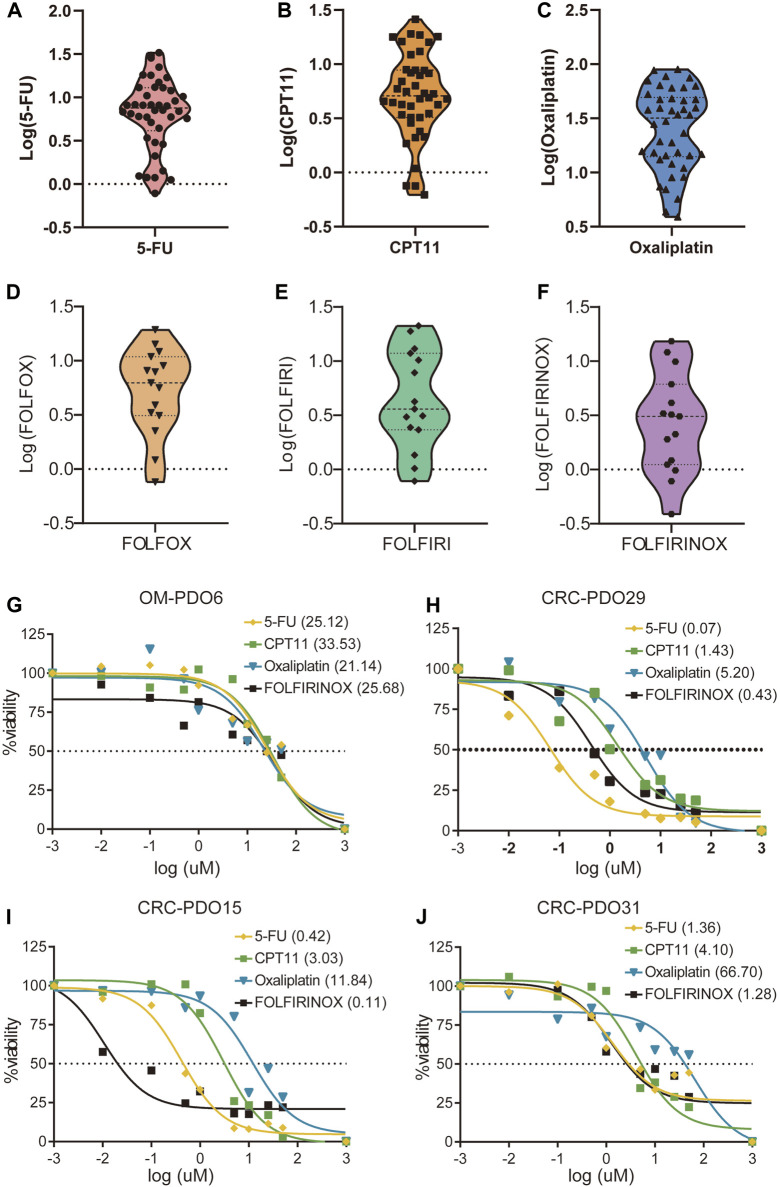
Drug responses of metastatic colorectal cancer organoid lines. Violin plot showing the distribution of the standardized IC50 values of the drugs, **(A)** 5FU, **(B)** CPT11 and **(C)** Oxaliplatin, in 30 mCRC organoids; Violin plot showing the standardized IC50 values of 15 mCRC organoids for **(D)** FOLFOX, **(E)** FOLFIRI, and **(F)** FOLFIRINOX chemosensitivity. *Ex vivo* chemosensitivity of four representative mCRC organoids, **(G)** OM-PDO6, **(H)** CRC-PDO29, **(I)** CRC-PDO15, and **(J)** CRC-PDO31, to 5-FU, CPT11, Oxaliplatin, and FOLFIRINOX presented in the form of standardized IC50s.

5-FU, CPT11, and oxaliplatin have been the most essential first-line chemotherapeutic drugs in the treatment of mCRC over the past decades. To evaluate the utility of mCRC organoid lines as a platform for assessing the drug response of primary tumors in patients, we performed *in vitro* drug sensitivity assays using PDOs. The drug sensitivity of the organoids was represented by the concentration that inhibited 50% (IC50) of the PDOs. 42 organoid lines were treated with 5-FU, CPT11, and oxaliplatin monotherapy, and 15 organoid lines were treated by the combination therapy based on the three drugs, including the FOLFOX, FOLFIRI, and FOLFIRINOX regimens. The *in vitro* chemosensitivity of PDOs to 5-FU, CPT11, or oxaliplatin monotherapy, as well as the combination therapies, are shown in the form of standardized IC50 values (Figures 4A–F). The median IC50 of mCRC PDOs was 9.68 μM (range from 0.07 μM to 32.75 μM) for 5-FU, 7.57 μM (range from 0.62 μM to 33.53 μM) for CPT11 and 33.56 μM (range from 3.90 μM to 89.68 μM) for oxaliplatin (Supplementary Table S3). The median IC50 of mCRC PDOs was 9.17 μM (range from 1.36 μM to 21.21 μM) for the FOLFIRI regimen, 8.53 μM (range from 0.76 μM to 23.12 μM) for the FOLFOX regimen and 5.31 μM (range from 0.11 μM to 25.68 μM) for the FOLFIRINOX regimen (Supplementary Table S4). Then we analyzed the sensitivity of four mCRC PDOs to single-agent and FOLFIRINOX regimens. OM-PDO6 was resistant to the three single agents and FOLFIRINOX, with an IC50 values ranging from 21.14 μM to 33.53 μM (Figure 4G). CRC-PDO29 was sensitive to the three single agents and FOLFIRINOX, with an IC50 values ranging from 0.07 μM to 5.20 μM (Figure 4H). However, CRC-PDO15 and CRC-PDO31 showed different chemosensitivities to single-agent and combination regimens (Figures 4I,J). Thus, the mCRC PDOs are heterogeneous in their chemoresponse to 5-FU, CPT11, and oxaliplatin doses.

### PDOs can reflect the previous treatment response of the corresponding patient

Previous studies have reported that the PDO drug screening test results correlated with the objective clinical response to chemotherapy (Chen et al., 2021; Hu et al., 2021; Mo et al., 2022). Here, we report data from five patients who received chemotherapy regimens of FOLFOX, FOLFIRI, and FOLFIRINOX, respectively. The chemotherapy responses were evaluated as resistant or responsive by radiography.

**FIGURE 5:**
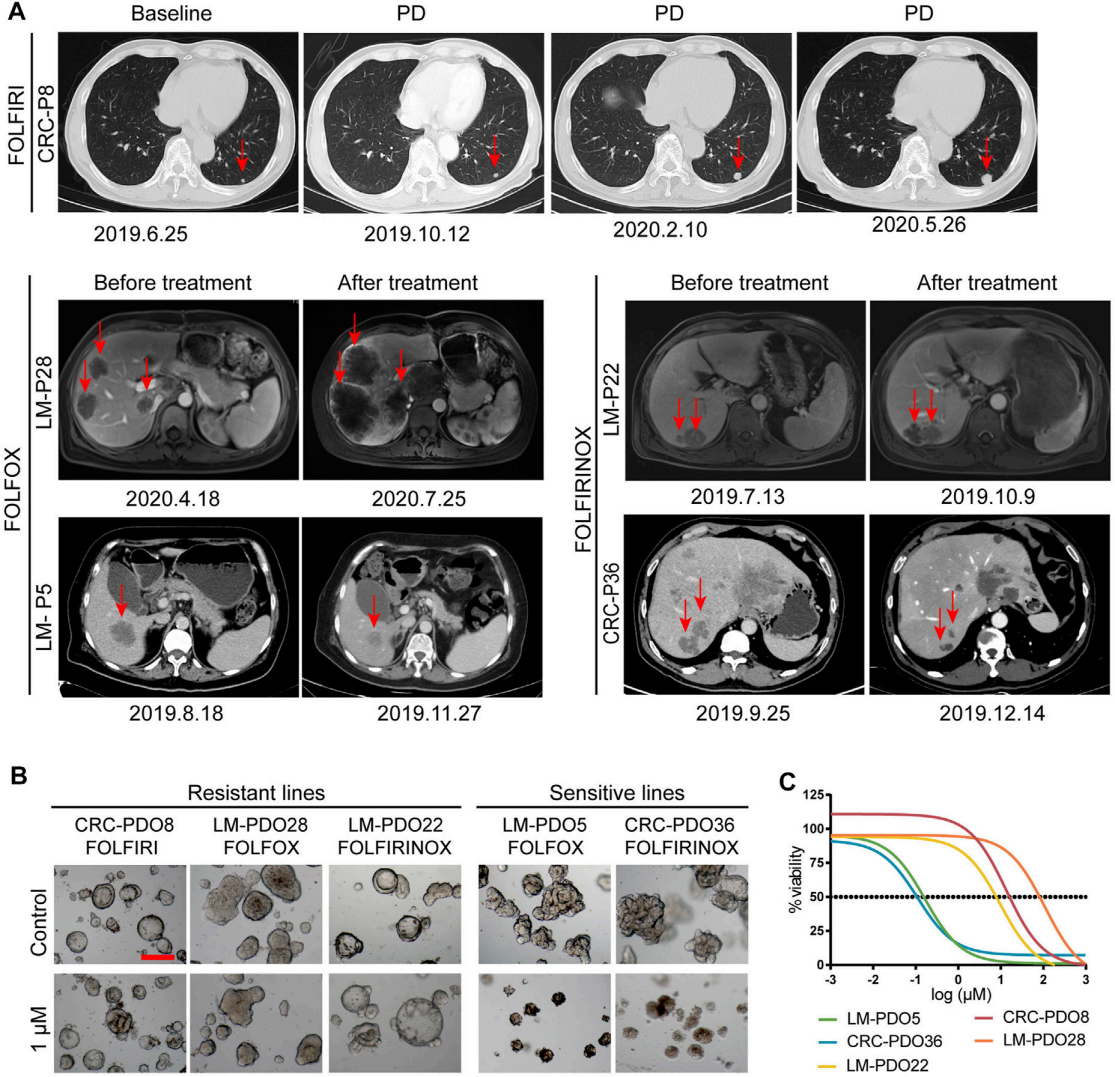
PDOs predict chemotherapy response of mCRC patients. **(A)** The imaging manifestations of target lesions in 5 mCRC patients before and after treatment, including the progression of lesions in CRC-P8, LM-P22 and LM-P28 patients and the regression of lesions in LM-P5 and CRC-P36 patients. **(B)** Representative bright-field images of organoids resistant to chemotherapy regimens, including CRC-PDO8, LM-PDO22, and LM-PDO28; Representative bright-field images of organoids sensitive to chemotherapy regimens, including LM-PDO5 and CRC-PDO36. **(C)** *Ex vivo* chemosensitivity of 5 PDOs in the form of dose-response curves were displayed. Scale bar, 200 μm. PD, progression disease.

As illustrated in Figure 5A, patient CRC-P8 was diagnosed with colorectal cancer with liver metastasis at the initial diagnosis and subsequently underwent enterohepatectomy, after which he received FOLFIRI chemotherapy. The patient showed resistance to this chemotherapy regimen and had a progressive disease in the lung. Then the lesions in the lung continued to progress during and after treatment. Patients LM-P28 and LM-P5 both received the FOLFOX chemotherapy regimen clinically. LM-P28 showed clinical resistance and rapid progression in tumor volume. At the same time, LM-P5 showed clinical sensitivity and regression in tumor volume. LM-P22 and CRC-P36 were patients diagnosed with CRC with multiple systemic metastases. They both received the chemotherapy regimen of FOLFIRINOX. LM-P22 showed clinical resistance, while CRC-P36 showed sensitivity to the FOLFIRINOX regimen. To determine whether the PDO pharmaco-phenotyping could represent the patients’ previous response to chemotherapy regimens, an *ex vivo* chemotherapy drug test was performed on PDOs derived from the corresponding patients.

Our data showed that CRC-PDO8, LM-PDO28, and LM-PDO22 were resistant to the FOLFIRI, FOLFOX and FOLFIRINOX regimens, respectively. Moreover, LM-PDO5 and CRC-PDO36 were sensitive to FOLFOX, and FOLFIRINOX regimens, respectively (Figures 5B,C). These results suggested that the data obtained from *in vitro* chemotherapeutic drug testing of PDOs can represent the clinical response to chemotherapy in the corresponding patients. Therefore, this preclinical model has a potential value for helping select the appropriate clinical chemotherapy regimens, which is critical for patients to avoid ineffective treatment due to unnecessary side effects, time consumption, and resource consumption.

## Data Availability

The raw data supporting the conclusion of this article will be made available by the authors, without undue reservation.

## Discussion

In the past decade, the prognosis of mCRC patients has significantly improved due to the increasing number of effective drugs (De Falco et al., 2020; Riedesser, Ebert, and Betge, 2022). However, the clinical outcome and treatment response of patients with CRC are still unsatisfactory (Linnekamp et al., 2015). At the same time, many drugs that have shown efficacy in tumor models ultimately fail in clinical trials or have poor efficacy in clinical patients, which indicates that there is a barrier between *in vitro* model systems and *in vivo* clinical practice (Ji and Wu, 2020). Therefore, preclinical models that accurately reflect the genetic diversity and specificity of tumors should be developed to help oncologists ensure that cancer patients are offered the optimal treatment options from the outset to avoid the potential side effects and unnecessary costs of ineffective treatments.

Organoids have revolutionized cancer research since 2009 (Sato et al., 2009) when organoid culture technology was discovered and applied to many other tumor types. Three-dimensional (3D) organoids have been used to model disease and development affecting most organs (Sato et al., 2009; Sato et al., 2011; Lau et al., 2020). To date, different culture methods have been developed to improve the efficiency of organoids (Fujii et al., 2016; Fujii et al., 2018; Pleguezuelos-Manzano et al., 2020). Researchers have successfully established many kinds of tumor organoid biobanks in the past decade (Broutier et al., 2017; Li et al., 2018; Yan et al., 2018; Kim et al., 2019; Kopper et al., 2019; Lohmussaar et al., 2021). In this study, we successfully established a living biobank containing 42 mCRC organoids derived from primary CRC and metastatic lesions. The overall success rate of 80.8% was almost the same as that reported previously (Ooft et al., 2019; Yao et al., 2020; Mo et al., 2022). We found that mCRC PDOs preserved the histopathologic features of the corresponding tumors, including biomarkers for clinical diagnosis and laboratory testing of CRC. Moreover, the *in vitro* chemosensitivity test revealed interpatient heterogeneity in the response of PDOs to monotherapy or combination therapy. Therefore, PDOs as tumor substitutes have the potential to be used for the development of personalized medicine.

Preclinical models have been widely used to discover new therapeutic agents and conduct preclinical trials of drug effectiveness (Chabner, 2016). Traditional cancer cell models are commonly used for high-throughput drug testing. However, these models fail to mimic 3D growth *in vivo*. In addition, such cancer cell lines might have undergone tremendous genetic alterations, so they cannot adequately recapitulate the biology of the corresponding original tumors (Li et al., 2008; Chabner, 2016). Although animal models are important for cancer research, their establishment requires a significant amount of time. Moreover, Moreover, due to the histological complexity and genetic heterogeneity of human cancers, animal models often do not truly reflect the pathogenesis of patients (Ji and Wu, 2020). Advances in the generation of PDO models will make it possible to establish a large biobank that more closely mirrors the original tumors, which can be adapted to identify sensitive agents and optimal chemotherapy regimens by drug sensitivity testing.

Various studies in recent years have shown that CRC PDOs can recapitulate patient responses in the clinic and may have predictive value (Verissimo et al., 2016; Vlachogiannis et al., 2018; Ooft et al., 2019). Here, we performed *in vitro* drug screening of PDOs to mimic clinical chemotherapy regimens. The FOLFIRI, FOLFOX, or FOLFOXIRI regimens are the most appropriate first-line chemotherapy options for advanced CRC patients (Colucci et al., 2005; Loupakis et al., 2014; De Falco et al., 2020), while the clinical response to these treatments varies among patients. In the present study, significant heterogeneity of drug response was observed in mCRC organoid lines, which is consistent with the diversity of responses to regimens in clinical practice, together with previous studies (Pauli et al., 2017; Li et al., 2019), This finding suggests that the patient-derived cancer organoid lines may become a valuable preclinical model for drug screening.

There were several limitations in our study. The lack of gene sequencing data for the organoids and their derived tumor tissue prevented us from comparing the genetic characterizations between them. The reasoning behind this notion is that previous studies (Sachs et al., 2018; Yao et al., 2020; Mo et al., 2022) have demonstrated that PDOs recapitulate the genetic characterization of parental tumors. The cutoff value of IC50 for organoids in the resistant and sensitive groups has not yet been defined, and further *in vitro* drug sensitivity tests of organoids combined with clinical data are needed for verification.

In summary, our results illustrate the potential use of PDOs as a promising new preclinical model to represent the characteristics of individual patients. The comprehensive characterization of mCRC organoid lines implies that they capture intra- and interpatient heterogeneity. This organoid model may play a potential role to be a valuable preclinical *ex vivo* model to predict patient responses to chemotherapy regimens. In the future, it will be necessary to conduct further research in clinical trials to determine whether the data from mCRC PDOs can be used to provide personalized therapy recommendations for patients in the advanced stage.
